# Predictors of Medical Students’ Perceptions of Psilocybin-Assisted Therapy for Use in Medical Practice

**DOI:** 10.7759/cureus.37450

**Published:** 2023-04-11

**Authors:** Karina Wang, Yiqun Sun, Brenda Nava, Luke Sampiere, Robin J Jacobs

**Affiliations:** 1 Dr. Kiran C. Patel College of Osteopathic Medicine, Nova Southeastern University, Fort Lauderdale, USA

**Keywords:** schedule 1 drug, legalization, medical education, palliative care, alternative medicine, psychiatric, mental health, magic mushrooms

## Abstract

Background

Psilocybin use, along with other psychedelics, has seen an increased interest among professionals in the medical community due to its potential therapeutic benefits for psychiatric disorders, substance use disorders (SUD), and palliative care. While it is certain that more research is necessary as psychedelic-assisted therapy becomes more prevalent, it will most likely be future physicians at the forefront of this neoteric care. Currently, physicians receive minimal training because of psilocybin’s contextual information and its current enlistment as a Schedule 1 drug per the United States Drug Enforcement Administration. Schedule 1 drugs, substances, or chemicals are defined as drugs with no currently accepted medical use and a high potential for abuse. As a rule, formal education on psilocybin is not included in medical school curricula, and very little is known about how medical students perceive it. The aim of this study was thus to assess current medical students’ perceptions of their knowledge, concern for possible negative effects, and perceptions about medical psilocybin to provide a deeper understanding of which factors may predict their overall perceptions of its future therapeutic use.

Methods

Medical students’ knowledge, concern for potential adverse effects, and perceptions of medical psilocybin were investigated using a cross-sectional survey study design. Data were collected in January 2023 from a convenience sample of United States medical students in years one to four of their program using a 41-item anonymous quantitative online survey. Multivariate linear regression modeling was performed to determine if perceived knowledge and beliefs about legalization would predict medical students’ attitudes about psilocybin use for therapeutic purposes.

Results

Two hundred and thirteen medical students completed the survey. Seventy-three percent (n=155) were osteopathic medical students (OMS), and 27% (n=58) were allopathic medical students (MDS). Regression modeling produced a statistically significant equation: (*F*(3, 13) = 78.858, *p* < .001), with an R^2^ = 0.573 (adjusted R^2^ = 0.567), indicating that greater (perceived) knowledge about medical psilocybin, less concern for its possible adverse effects, and greater belief in the legalization of psilocybin for recreational use significantly contributed to positive perceptions of psilocybin use in medical practice.

Conclusions

In this sample, medical students with greater self-assessment of their knowledge about medical psilocybin, less concern for its potential adverse effects, and more positive views about recreational psilocybin legalization predicted positive attitudes about its medical use. Interestingly, although some participants had positive perceptions about the legalization of psilocybin for medical use, endorsing its legalization for recreation was related to greater positive attitudes toward medical psilocybin in general, a finding that appears to be somewhat counterintuitive. More research is warranted to further explore medical trainees’ attitudes toward it, as psilocybin is a promising therapeutic intervention. If medicinal psilocybin continues to gain attention among patients and physicians alike, it will be imperative to evaluate its therapeutic efficacy, proper use, dosing, and potential for negative effects, in addition to preparing students to endorse therapeutic psilocybin when warranted.

## Introduction

There has been a growing interest in the use of psilocybin (an active ingredient found in various species of mushrooms that contain psychoactive properties and are colloquially termed "magic mushrooms") in medicine due to its potential therapeutic benefits for patients with psychiatric disorders, substance use disorders, and those in need of palliative care. Psychedelics are hallucinogenic drugs whose primary purpose is to trigger extraordinary mental states and/or an expansion of consciousness. Termed a psychedelic "renaissance," the beneficial use of psychedelics such as psilocybin, ketamine, and methylenedioxymethamphetamine (MDMA) has recently regained attention [1,2]. Psilocybin is an active ingredient in various species of mushrooms, often referred to as "magic mushrooms." Although classified as a Schedule 1 drug (having no medically accepted use and a high abuse potential), the Food and Drug Administration (FDA) has only just acknowledged that psilocybin may have substantial benefits over current treatment options, granting it "breakthrough therapy designation" [3].

Psilocybin-assisted therapy combines the pharmacologic effects of psilocybin with psychological support. As an adjunct treatment, psilocybin’s efficiency has been used in studies about mental health disorders, alcohol use disorder (AUD), tobacco cessation, and depression in individuals with life-limiting illnesses [4-7]. Findings from studies suggest that psilocybin may be effective in producing fast-acting and long-lasting antidepressant effects in patients with major depressive disorder [8]. Moreover, psilocybin treatment under psychologically supported conditions has been shown to significantly relieve severe mood disorders (e.g., anxiety, depression) in individuals with deadly cancers [9,10].

Adverse effects

While some studies report that recreational psilocybin demonstrates some negative consequences, such as physical aggression to the point of seeking medical attention, there have been no serious harmful events of psilocybin-assisted therapy reported [5,9]. Taking this into consideration, psilocybin may serve as a promising therapeutic intervention. Therefore, the importance of therapeutic preparation and the awareness of psychotherapeutic, pharmacological, and contextual information is needed for the future healthcare provision of psilocybin therapy [11,12].

Attitudes and knowledge about psilocybin

Psychiatrists and other mental health care providers, as well as university students, have suggested that psilocybin may be useful in treating certain psychiatric disorders. In a study of 324 psychiatrists, 42% reported that psychedelics can be a therapeutic tool for psychiatric disorders [13]. Similar results were seen in a study of 124 college students, where 35% thought psilocybin might be a beneficial therapy for depression and 39% for anxiety [14]. Palliative care providers support the idea that psychedelic-assisted therapies have the potential to fulfill the current gap in treating refractory existential distress [15]. Nurses and mental health nurse practitioners, who play an integral role in psychedelic-assisted therapy, have also recognized that their field must increase its awareness of psilocybin’s rising therapeutic benefits. To safely plan their involvement with psychedelic-assisted therapies, there has been a call for interdisciplinary collaboration among researchers, scholars, and clinical experts to further study psilocybin’s therapeutic potential, proper use, limitations, and adverse effects [11,16].

There are only a few studies to our knowledge that pertain to healthcare workers' perceptions, knowledge, and concerns about psilocybin-assisted therapies. Some studies indicate that mental health practitioners have neutral attitudes and low self-rated knowledge about psilocybin [17]. Young psychiatry residents reported less concern about hallucinogens’ adverse effects as well as greater optimism for their therapeutic potential [13]. Palliative care providers expressed that psychedelic-assisted therapies have powerful potential within their field [15]. Moreover, psychologists reported favorable attitudes but have raised concerns about possible psychiatric and neurocognitive effects due to a lack of knowledge about psilocybin’s therapeutic benefits [18]. Regarding patient attitudes, one study that investigated the perceptions of patients under the care of a mental health provider reported that 25% of the participants reported being knowledgeable about psilocybin, 54% noted they would agree to psilocybin therapy if a physician recommended it, and 55% would be inclined to down-titrate from prescription medications [19]. Most of the demographic groups listed in the respective study favored further psilocybin-assisted therapy research.

Legalization

This current uptick of decriminalization and legalization has been in response, in part, to current psilocybin research and its promising results. National and state governments have begun to decriminalize and legalize psilocybin use for medicinal purposes. Australia has officially recognized psilocybin as a medication for treatment-resistant depression (TRD) [20]. Oregon became the first U.S. state to both decriminalize and legalize psilocybin for therapeutic use, with Colorado shortly following [21]. In addition, Texas was the first state to fund a psychedelic medicine trial that investigated the use of psilocybin in war veterans affected by post-traumatic stress disorder (PTSD) [22]. Several other U.S. cities, including Michigan, Massachusetts, Washington, California, and Washington D.C. have also recently decriminalized psilocybin [23]. However, there are still regulations and restrictions about psilocybin’s legality in each of its respective locations.

The emerging research and interest in psilocybin and its potential as a treatment for certain psychiatric disorders precipitated the implementation of this study, which investigated the perceptions, knowledge, and concerns about potential adverse effects of psilocybin among medical students, who, as future physicians, will have a role in its clinical integration and interpretation.

Research questions

The goal of this research was to answer the following questions: 1) What are the perceptions, perceived knowledge, and concerns of medical students regarding psilocybin being used medically? 2) Will perceptions of one’s knowledge about psilocybin, concern for its possible adverse effects, and attitudes toward the legalization of psilocybin make statistically significant contributions to the overall perceptions of psilocybin in medical students?

Hypotheses

This study sought to test the hypothesis that medical students’ perceptions of their medical psilocybin knowledge, concern for possible adverse effects of its use, belief in legalizing psilocybin for medical use, and belief in legalizing psilocybin for recreational use will make statistically significant contributions to their overall attitudes toward psilocybin use for medical purposes.

## Materials and methods

This cross-sectional survey study investigated medical students’ knowledge and attitudes about psilocybin being used for medical purposes. Data were collected using an online survey distributed via student email listservs. This study was approved by the Nova Southeastern University Institutional Review Board (Protocol No. 2022-572).

Sample and survey distribution

A cross-sectional survey study was conducted in January 2023 using an anonymous online survey administered via email. A convenience sample of 1,447 osteopathic medical students in one Florida medical school plus approximately 90 allopathic medical students across the United States of America was used to collect data. The survey was created using REDCap (http://projectredcap.org/), an application for creating and managing online surveys. Informed consent was assured by providing a letter or participation explaining the study in detail, including the option to not participate, that the survey was anonymous, and that participating (or not participating) in the study would not affect their grades or academic standing. In addition, a link to the survey was provided that stated, "By clicking this link, I consent to participate in this study." The assessment instrument took about five to seven minutes to complete. Several update emails were sent as reminders at predetermined intervals to encourage participation.

The instrument

The researchers developed the survey instrument after reviewing recent and relevant studies published in peer-reviewed journals. The 41-item survey assessed participants’ perceived knowledge, concerns, and perceptions about medical psilocybin. The survey used Likert-type items with a six-point response scale (1=strongly agree to 6=strongly disagree), categorical items, and yes/no questions. Many of the items were adapted from various published studies, mostly regarding the use of medical cannabis as no measures specific to medical psilocybin could be found [7,24-29]. The content areas assessed are delineated below.

Perceived Knowledge of Psilocybin for Medical Use (Scale)

To assess perceived knowledge of medical psilocybin, four Likert-type items were included that assessed participants’ perceived knowledge about the medical uses of psilocybin. The items were rated using a six-point response scale (1=strongly agree to 6=strongly disagree) and included: 1) "I am familiar with the possible therapeutic effects of psilocybin." 2) "I have substantial knowledge about psilocybin." 3) "I am extremely confident regarding my current knowledge of psilocybin." 4) "I have good knowledge of the side effects of psilocybin." The items for this scale were adapted from Jacobs and colleagues’ study on perceptions of medical psilocybin among medical students [25,26].

Concern for Possible Adverse Effects of Medical Psilocybin (Scale) 

Adapted from Jacobs and colleagues’ study were four items to assess participants’ concern for possible adverse effects of psilocybin use: 1) "I am concerned with psilocybin’s potential for addiction or its psychoactive properties." 2) "I am concerned about the potential side effects of psilocybin use." 3) "I am concerned with psilocybin’s potential for abuse or misuse." 4) "Psilocybin use can be addictive." [25,26].

Perceptions About Medical Psilocybin (Scale)

Fourteen items assessed overall perceptions regarding medical psilocybin. Sample items on the scale are: 1) "I believe that psilocybin is a legitimate medical therapy." 2) "I am certain about psilocybin’s therapeutic value." 3) "Psilocybin helps patients who suffer from chronic, debilitating medical conditions." 4) "Using psilocybin poses serious mental health risks." 5) "Using psilocybin poses serious physical health risks, as adapted from Jacobs and colleagues." [25,26].

Utility of Psilocybin for Certain Medical Conditions

The survey included an item asking participants if they thought that psilocybin was therapeutic for any or all of the 26 medical conditions or symptoms (e.g., fibromyalgia, glaucoma, nausea), mental health conditions (e.g., mood disorders), or health behaviors (e.g., smoking) listed, with an option for “other.”

Single Items

Singe items developed by the researchers were included that collected data on demographic information (i.e., age, current U.S. state of residence, political views (i.e., conservative, liberal, independent), whether they were allopathic or osteopathic medical students, year in the program, personal use of or knowledge of someone who has used recreational and medical psilocybin, and opinions about if recreational and medical psilocybin should be legalized. Participants were also asked to report whether they had been instructed about psilocybin while in school and if they were interested in learning about psilocybin.

## Results

Preliminary analysis

A descriptive, cross-sectional study was conducted in January 2023 using an anonymous online survey administered via email to the 1,447 osteopathic medical students in a large medical school in South Florida and approximately 70 allopathic medical students via snowball sampling methods throughout the United States (total: 1,517).

Of the 1,517 students enrolled in a medical school program from years one to four, 340 returned the survey (22% response rate). Of those, 127 cases were omitted due to: 1) having less than 66% of the survey items answered (n=108), 2) being left completely blank after opening the link (n=11), or 3) having reported: "I am not a medical student" (n=8), leaving 213 surveys for the final analyses (63% completion rate).

IBM SPSS Statistics for Windows, Version 28.0 (Released 2021; IBM Corp., Armonk, New York, United States) [30] was used for the analyses after downloading the data from REDCap [31]. Visual inspections of the observed distributions and tests for skewness and kurtosis (i.e., assessments of normal distributions) were performed. For this sample, reliability estimates (i.e., Cronbach’s alpha) for the three measures in the survey were computed: perceived knowledge of medical psilocybin (α = .92); concern for possible adverse effects (α = .88); and perceptions of psilocybin use in medicine (α = .92). All reliability estimates were within acceptable limits (α >.70) [32].

Before performing the regression, multicollinearity testing was conducted. The independent variables were within acceptable variance and inflation factor limits. Moreover, the scales were normally distributed (i.e., the data did not deviate from normality enough to affect inference).

Data analysis

For discrete variables, sample characteristics are reported as frequency and percentage. Continuous variables are reported as means and standard deviation (SD). Point-biserial correlation and multivariate linear regression were used for hypothesis testing.

Descriptive statistics

Sample Characteristics

The mean age of the participants was 26 years (range: 19 to 53 years). Seventy-three percent (n=155) were osteopathic medical students (OMS), and 27% (n=58) were allopathic medical students (MDS). Information regarding the sample characteristics is in Table 1.

**Table 1:** Characteristics of the sample (N=213)

Characteristic	n	%*
Race		
White	136	63.8
Black	14	6.6
American Indian	1	0.5
Asian or Pacific Islander	42	19.7
Prefer not to answer	13	6.1
Missing	206	96.7
Identify as Hispanic/Latinx	28	13.1
Sex		
Female	122	57.3
Male	78	36.6
Non-binary/Prefer not to answer	13	6.1
Year in Medical School Program		
First year medical student	70	32.9
Second year medical student	77	36.1
Third year medical student	35	16.4
Fourth year medical student	31	14.6
Political Identification		
Conservative	22	10.3%
Liberal	109	51.2%
Independent	43	20.2%
Other	15	7.0%
Prefer not to answer		
Used psilocybin recreationally	70	32.9
Used psilocybin for medical use	19	8.9
If used psilocybin for medical use, was under the supervision of a trained health professional	5	2.3
Thought that psilocybin should be legal for recreational use	176	82.6
Thought that psilocybin should be legal for medical use	110	51.6
Has desire to learn more about psilocybin	193	90.6
Psilocybin is taught in-depth as part of my medical school training		
Strongly agree	5	2.3
Agree	6	2.8
Somewhat agree	10	4.7
Disagree	20	9.4
Strongly disagree	81	38.0

Participants from 22 states and the District of Columbia (D.C.) completed the survey. Most of the participants (n=142; 66.7%) reported they currently reside in Florida, followed by Massachusetts (n-19; 8.9%) and California (n=15; 7.9%). Table 2 reports the frequencies for the residence of the participants. 

**Table 2:** U.S. states (including Washington D.C.) in which the participants reside

	N	%
Alabama	1	0.5%
Arizona	1	0.5%
California	15	7.0%
Colorado	1	0.5%
Connecticut	1	0.5%
Florida	142	66.7%
Georgia	2	0.9%
Hawaii	1	0.5%
Illinois	1	0.5%
Louisiana	1	0.5%
Maryland	3	1.4%
Massachusetts	19	8.9%
Michigan	1	0.5%
Missouri	1	0.5%
New York	1	0.5%
North Carolina	1	0.5%
Pennsylvania	4	1.9%
Tennessee	1	0.5%
Vermont	1	0.5%
Virginia	1	0.5%
Washington	1	0.5%
Wisconsin	1	0.5%
Washington, D.C.	3	1.4%
Prefer not to answer	9	4.2%

Medical Uses of Psilocybin

**Figure 1:**
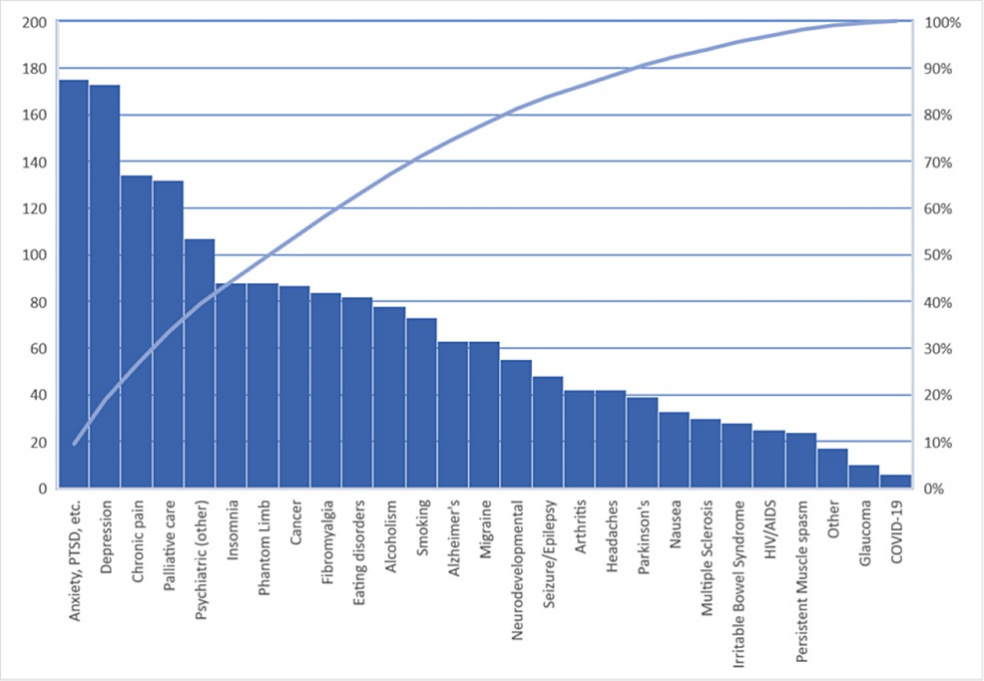
Participant responses to item “Psilocybin is effective for the following medical conditions (check all that apply)”

Figure 1 demonstrates the participants’ opinions of the therapeutic uses of psilocybin for selected medical conditions, symptoms, and health behaviors.

Major study variables

Measures of Variability

Summary statistics (means, standard deviation, and range) for major study variables (perceived knowledge, concern, and perceptions) using scales with a six-point Likert-type response set (1=strongly agree to 6=strongly disagree) are provided in Table 3.

**Table 3:** Survey measurement outcomes Mean represents the mean score for any item on a scale, and SD indicates the standard deviation. For the perceived knowledge scale and the psilocybin perceptions scale, higher scores indicate greater perceived knowledge and more positive perceptions of medical psilocybin. For the concern for possible adverse effects, a lower score indicates greater concern. The single items about legalization were dichotomous, (where 1=yes and 2=no). Range indicates the Likert-type scale score range for each item on the scale.

Scale	n	Mean (SD)^a^	Range^b^
Perceived knowledge of psilocybin	213	3.65 (1.32)	1-6
Concern for possible adverse effects	212	3.29 (1.14)	1-6
Perceptions of medical psilocybin	213	2.46 (0.78)	1-6
Should be legalized for recreational use	206	1.59 (0.71)	1-2
Should be legalized for medical use	206	1.23 (0.59)	1-2

Opinions About the Legalization of Psilocybin

Most of the participants stated that psilocybin should be legalized for recreational purposes (n=176; 82.5%), yet only half of the participants (n=110; 51.6%) thought that psilocybin should be legalized for medical purposes.

Correlations

Table 4 reports the results of the point-biserial correlation analysis for preliminary hypothesis testing. All correlations were statistically significant, p < .05.

**Table 4:** Correlations between perceptions of medical psilocybin and major study variables **. Correlation is significant at the 0.01 level (2-tailed). *. Correlation is significant at the 0.05 level (2-tailed).

	Perceptions of medical psilocybin
Perceived knowledge of psilocybin	.610**
Concern for possible adverse effects of psilocybin	-.599**
Psilocybin should be legalized for recreational use	.382**
Psilocybin should be legalized for medical use	.330**

Regression model

Table 5 reports the results of the multivariate linear regression analysis that was performed to identify variables that predict medical students’ perceptions of medical psilocybin. The model produced a significant regression equation: (F(3, 13) = 78.858, p < .001), with an R2 = 0.573 (adjusted R2 = 0.567). Greater perceived knowledge about medical psilocybin, less concern over possible adverse effects of psilocybin use, and the belief that psilocybin should be legalized for recreational use was associated with more positive perceptions about medical psilocybin. The belief that psilocybin should be legal for medical purposes was not a statistically significant predictor in the model. The percentage of variance in the scores accounted for by the model was 57%. The combination of predictor variables representing modifiable behaviors (i.e., knowledge, concerns) predicting even a moderate amount of the variance could have a significant implication for guiding practice.

**Table 5:** Multivariate linear regression model for "perceptions of medical psilocybin" Predicted variable: perceptions of medical psilocybin *p < 0.05, **p < 0.01

	Unstandardized B	Standard error	Beta (β)	t-statistic	Significance (p)
(Constant)	21.242	4.167		5.097	0.000**
Perceived knowledge of medical psilocybin	1.005	0.120	0.488	8.380	0.000**
Concern for possible adverse effects of psilocybin	-0.575	0.155	-0.245	-3.720	0.000**
Belief in decriminalization (legalization) of psilocybin for recreational use	4.258	1.300	0.192	3.275	0.001

## Discussion

This study provided some insight into the perceptions and knowledge of psilocybin’s potential therapeutic uses among a sample of medical students. There were significant associations between participants’ knowledge of psilocybin and their attitudes towards its use as a medical therapy. The study also supports findings from previous literature regarding medical trainees and those professionals in the early stages of their careers who report favorable attitudes towards psychedelic therapies as well as higher-rated self-knowledge of psilocybin. Participants’ opinions towards recreational use were also studied, along with their concerns regarding potential negative effects.

Previous literature has shown that overall neutral attitudes towards psilocybin exist among mental health professionals who also report low self-rated knowledge of psilocybin. The average age of participants in this study was 47 years [17]. A survey among clinical psychologists with an average age of 50 years also indicated a lack of psychedelic understanding [18]. Given these findings, it is logical to assume that it may be difficult for patients to access psycho-integrative treatment since knowledge is one of the biggest barriers to professionals recommending novel forms of treatment [33]. This lack of knowledge can be greatly attributed to the paucity of psilocybin training in medical school curriculums. In this study, nearly half of the participants reported that they had not received in-depth training on psilocybin during medical school, while nearly all (90.6%) claimed to want more psilocybin education in medical school.

Attitudes among younger professionals and professionals-in-training have proven to be significantly more positive compared to their older counterparts [13]. Our study supports these findings; the results obtained from our survey showed medical students with a greater self-assessment of their knowledge about medical psilocybin had positive perceptions of psilocybin use in medical practice. These findings parallel multiple studies done on different population groups given that the mean age of the participants in this study was 26 years. One study on college students’ perceptions regarding hallucinogenic drugs found that of the students who reported being knowledgeable about hallucinogenic drugs, 43.5% believed that hallucinogens could be safely used recreationally, compared with 38.2% of the overall sample [14]. In separate studies, mental health service users [19] and early-stage psychiatric trainees [13] found that younger users reported more favorable attitudes toward the potential therapeutic use of psychedelics. These findings suggest that the younger generation and early-stage medical trainees align with the growing interest in psychedelic therapy. It could be suggested that medical educators incorporate psilocybin education as it relates to clinical decision-making [17,33]. This may pave the way for more research into future psychedelic treatments for mental health and palliative care, given that practice informs research and vice versa.

Regarding medicinal psilocybin’s potential adverse effects, participants in this study reported low levels of concern. This finding seems to follow the same age and training-stage trend as with knowledge of psilocybin. In previous studies, most psychiatrists viewed hallucinogens as potentially hazardous, though the minority, composed of younger trainees, reported less concern for their risks [13]. Clinical psychologists have also raised concerns about possible neurocognitive and psychiatric risks [18]. Moreover, palliative care providers, mostly between the ages of 36-65, have raised concerns about current medical stigma. There were some fears noted for possible triggers of relapse in patients with a history of substance abuse disorders and for the possibility of lasting psychological harm. However, providers have expressed confidence in psychedelic-assisted therapy within properly controlled settings [15].

The results of this study also indicated a greater belief in the federal legalization of psilocybin for recreational purposes than for medical purposes, a finding that may seem counterintuitive given the demographic of medical students. This may be a result of the general increase in recreational psilocybin and the negative press regarding it and other street drugs targeted at younger populations. However, the findings of this study are consistent with those found among mental health service users [34,35] and providers, who also reported positive attitudes regarding the decriminalization of psilocybin [17]. In previous studies, only 38.2% of college students agreed or strongly agreed that hallucinogenic drugs could be safely used recreationally. These differing results could be attributed to the fact that the only college students surveyed were in the Midwest [14], whereas the current study included only four participants from that region. It should also be noted that of the 50.8% of college students who self-reported knowledge about hallucinogenic drugs, 43.5% agreed that hallucinogenic drugs could be safely used recreationally.

Moreover, the connection between our survey results, osteopathic medicine, and insufficiently treated existential distress in palliative care shares a fair amount of common ground. A unique distinction of osteopathic medicine principles is the body-mind-spirit paradigm of health, a holistic principle adapted from Native American healing principles [36]. Many of the participants were osteopathic medical students who are trained under this body-mind-spirit approach to healthcare, and positive perceptions about psilocybin may be attributed to this principle. Furthermore, it is important to understand psilocybin’s history and purpose within indigenous communities for spiritual healing [37]. The goal of psilocybin utilization within those communities was not to break down the perception of reality but rather to facilitate psycho-integration for therapeutic purposes [38]. Interestingly, palliative care providers state that a psychosocial-spiritual approach is needed to tackle the insufficient treatment of existential distress, elaborating that existential distress is de-emphasized within medical training due to its conflict with current medical culture. Currently, there is an increasing number of medical providers who believe that psychedelic-assisted therapy could hold promise in addressing this gap [15].

Limitations

There are several limitations to this study. First, through convenience and snowball sampling recruiting methods, the true distribution of the medical student population could not be gauged. An increased risk of sample bias and margin of error is likely since participants tend to refer to people who are like themselves. Because most of our participants were Florida-based osteopathic medical students, medical students from other regions were not fully represented, making it difficult to generalize the results to all medical students in the United States of America. Moreover, it was impossible to ascertain if participants differed from nonparticipants as the survey was anonymous. Second, about half of the participants identified politically as "liberal," which is slightly higher than the national percentage, and about one-third of the participants reported using psilocybin recreationally. Third, a limitation may have been the exclusivity of questions solely on psilocybin and not on other psychedelics; the notion of psilocybin as therapy may have been confused with other hallucinogenic drugs and thus may have skewed responses. Last, cause-and-effect relationships or changes over time cannot be predicted using a cross-sectional survey design such as the one used in this study. Given the aforementioned limitations, results generated from this study should be taken with caution.

## Conclusions

The results of this study show that medical students with a greater self-assessment of their knowledge about medical psilocybin, less concern for its potential adverse effects, and the opinion that psilocybin should be federally legalized for recreational purposes have more positive attitudes about its medical use. A finding that appears counterintuitive showed that although some participants had positive perceptions about the legalization of psilocybin for medical use, those endorsing its legalization for recreational use had greater positive attitudes. Our results add to other bodies of literature regarding perceptions of different populations on psychedelics, further showing more support for continuing medical exploration and research on psilocybin’s potential therapeutic use for psychiatric disorders and palliative care. More research is necessary to further explore medical trainees’ attitudes towards psilocybin as a promising therapeutic intervention as medicinal psilocybin continues to gain attention among patients and physicians alike. As current medical students will soon become the gatekeepers for patient access to psilocybin treatment, it will be imperative to evaluate psilocybin’s therapeutic efficacy, proper use, dosing, and potential for adverse effects. In addition, increasing students’ readiness and knowledge to provide medicinal psilocybin as a viable treatment when warranted will ensure the prioritization of therapeutic outcomes and lead to greater benefits for society and the mental health professional community.
